# An Integrated Control Plan in Primary Schools: Results of a Field Investigation on Nutritional and Hygienic Features in the Apulia Region (Southern Italy)

**DOI:** 10.3390/nu13093006

**Published:** 2021-08-28

**Authors:** Vincenzo Marcotrigiano, Giacomo Domenico Stingi, Simona Fregnan, Pantaleo Magarelli, Pietro Pasquale, Samuele Russo, Giovanni Battista Orsi, Maria Teresa Montagna, Christian Napoli, Christian Napoli

**Affiliations:** 1 Food Hygiene and Nutrition Service, Local Health Unit BT, Department of Prevention, 76125 Trani, Italy; giacomodomenico.stingi@aslbat.it (G.D.S.); pantaleo.magarelli@aslbat.it (P.M.); 2 Clinic for Diabetology and Endocrinology, Unique District, Local Health Unit BA, 70121 Bari, Italy; simona.fregnan@asl.bari.it; 3 Apulia Region, Health and Wellness Promotion Section, Food Hygiene and Preventive Nutrition Organizational Structure, 70126 Bari, Italy; p.pasquale@regione.puglia.it; 4 Department of Computer, Control, and Management Engineering, Sapienza University of Rome, 00185 Rome, Italy; samuele.russo@uniroma1.it (S.R.); cnapoli@diag.uniroma1.it (C.N.); 5 Department of Public Health and Infectious Diseases, Sapienza University of Rome, 00185 Rome, Italy; giovanni.orsi@uniroma1.it; 6 Department of Biomedical Science and Human Oncology, University of Bari Aldo Moro, 70124 Bari, Italy; mariateresa.montagna@uniba.it; 7 Department of Medical Surgical Sciences and Translational Medicine, Sapienza University of Rome, 00189 Rome, Italy; christian.napoli@uniroma1.it

**Keywords:** nutritional knowledge, eight-year-old students, health promotion, official control, school canteen

## Abstract

Data concerning overweight and obesity in children and adolescent populations are alarming and represent one of the most serious public health problems of our time. Moreover, it is demonstrated that the school environment may play an important role in health promotion with regard to nutritional aspects. This article reports the results of a study conducted in the Apulia region (Southern Italy), aimed at providing an integrated surveillance of the behaviors related to nutrition habits in students and the hygienic and nutritional conditions of the school’s canteens attended by enrolled students. To this purpose, a sample of 501 students attending primary school (third class—children approximately eight years old) replied to a validated questionnaire, and official controls (OC), of both food and nutritional safety, were performed in 22 primary schools. A team of healthcare professionals carried out the study, and the implementation of all the prescribed improvement actions were subsequently verified through follow-up OC. The results of our study show a critical situation in the student sample, with 41.3% of children having a weight excess (overweight or obesity). With regard to the children’s behaviors, only 59.8% of children ate at least one fruit or had a fruit juice for breakfast, and 10.8% did not have breakfast at all. Overall, 40.1% of the total children played outdoors the afternoon before the survey and 45% reported going to school on foot or by bicycle. During the afternoon, 83.5% of the sample watched television or used video games/tablets/mobile phones, while 42.3% played sports. The schools had an internal canteen with on-site preparation of meals in 36.4%, the remaining 63.6% received meals from external food establishments. With regard to OC, for the hygienic–sanitary section, eleven prescriptions were issued, in the great part related to the structure and organization of the canteen. For the nutritional section, nine corrective actions were prescribed, mainly related to official documents and management. The follow-up OC showed that all prescriptions were subsequently addressed. Eating at school was less frequent among obese and overweight students compared with those with normal weight. Although this evidence needs to be further confirmed, it highlights the potential role that the school canteens may play in health promotion and prevention of nutritional disorders. On the other hand, in order to fulfill its health promotion task, the school canteens have to comply with official regulations and guidelines; therefore, OC during the management of the food service at school are needed.

## 1. Introduction

The spread of non-communicable diseases (NCDs), such as obesity, has become a paramount concern in the world health panorama. In order to limit the spread of such diseases, the World Health Organization (WHO) has identified different strategic areas of intervention for health promotion [1]; however, such actions first require a precise assessment of the state of health in the target population. In Italy, where obesity and overweight have been identified among the priority areas for intervention, a national surveillance project for nutritional status assessment and related factors in children at the third year of primary school started in 2008 [2]. The last survey was conducted in 2019 and showed that 20.4% of eight-year-old children were overweight, while 9.4% were affected by pathological obesity [3]. In particular, males were more affected, especially in the southern regions of Italy, as well as in families with a disadvantaged economic status [3].

The selected age group (eight-year-old) is considered a main target for prevention; in fact, the choices, attitudes, and behaviors adopted at this age have life-long repercussions and can be further amplified by other factors, especially in the adolescent period [4]. An international study conducted in 2018, investigated the Italian population aged 11, 13, and 15 years. This latter study highlighted that 16.6% of the subjects of this group were overweight, while 3.2% were obese. Overweight and obesity seem to affect males more often than females, and there is a confirmed higher prevalence in the southern regions than in the central–northern ones [4]. These data are also in line with the nutritional status among university students: a study in southern Italy reported a 17.8% and 3.4% prevalence of overweight and obese students, respectively [5].

Comparison with international data shows that weight excess is more common in Italian youth (in all age groups) with respect to the European average [6]. In addition, the COVID-19 pandemic also worsened this situation, aggravating lifestyle habits, limiting physical activities, and creating psychological distress [7,8].

It is also demonstrated that nutritional status in school-aged children is related to unhealthy behaviors (such as skipping breakfast, low intake of fruits and vegetables, and unhealthy snacking) and insufficient physical activity, which may be reflected in cognitive performance too [9,10]. In Italy, with regard to these aspects, the last national survey on nutritional aspects showed that 44.3% of children skip or have an inadequate breakfast, 24.3% do not eat fruit or vegetables daily, unhealthy snacks are consumed by 48.3%, and 20.3% do not practice any physical activity [3]. At the same time, the increasing prevalence of childhood overweight and obesity is often related to school environment features [9].

Children spend six or more hours per day at school for more than six months per year; therefore, an education intervention focused on improving nutrition knowledge, attitudes, and practices among primary school children must also involve the food quality available at school canteens. Health promotion interventions for school canteens, aimed at offering healthier food, seem promising and effective in improving the school food environment [11].

In light of these data, the Apulia region has identified schools to be one of the most important settings for health promotion, also from a nutritional point of view. Therefore, starting from the forecasts of the Regional Prevention Plan (PRP) 2010–2013 with the “School in Health” program, a “Regional Strategic Plan for Health Promotion in Schools: Catalogue” is also periodically issued [12,13]. Subsequently, the 2014–2018 PRP further focused its attention on the need to also provide guidelines on nutritional safety [14]. Therefore, the Regional Decree no. 1435 of 2 August 2018 has drawn up the “Guidelines for School and Corporate Catering” to ensure the adoption of correct eating habits for health promotion and disease prevention, fighting against an incorrect diet [15].

Starting from the new regional guidelines, this article shows the results of an integrated project aiming to (i) evaluate anthropometric data and behaviors in terms of nutrition habits in a sample of students attending primary school (third class—children approximately eight years old); (ii) carry out, in the same schools, the official controls (OC) in terms of both hygiene conditions and nutritional normative aspects foreseen by the DGR no. 1435 of 2 August 2018; and (iii) verify, through follow-up OC, the implementation of all the prescribed improvement actions.

## 2. Materials and Methods

The study was conducted in the Province BAT during the 2018–2019 school year (September 2018–June 2019) by the Food Hygiene and Nutrition Service (SIAN) of the Prevention Department of the Local Health Unit (LHU). The investigation was performed in accordance with the World Medical Association Declaration of Helsinki and did not include any experiments involving human or biological human samples, nor research on identifiable human data. Regardless, the study protocol was approved, also with regard to ethical issues, by the Regional Sanitary Authority (approval no. 238_2018).

### 2.1. Behaviors in Terms of Nutrition and Anthropometric Data

A reduced and adapted version of the questionnaire used during the 2016 national surveillance study was used in order to evaluate behavior with respect to nutrition habits, as well as to evaluate anthropometric data, in a sample of students attending the third year of primary school (children 7 to 9 years of age) [16,17]. The body mass index (BMI) was calculated and subsequently expressed as a percentile obtained with respect to age-and sex-related growing reference data provided by WHO for the European region [18]. In order to classify children in terms of weight status categories, the reference percentile ranges provided by the US Centers for Disease Control and Prevention (CDC) were used [19]: underweight (less than the 5th percentile); normal or healthy weight (from the 5th percentile to less than the 85th percentile); overweight (from the 85th to less than the 95th percentile); obese (equal to or greater than the 95th percentile). Additional factors such as general information on physical activity and use of mobile devices were also investigated. The proposed version of the questionnaire was validated during a pilot study in a sample of 100 eight-year-old students (data not reported or included in the study). In the same pilot sample, the reliability index was assessed using Cronbach’s alpha (internal consistency coefficient): the alpha value achieved was 0.79, showing a satisfactory level of reliability [20]. Moreover, the modified version of the questionnaire was also validated during the pilot study in terms of intelligibility: the students were asked to assign a score to each item of the questionnaire on a 7-point-scale (from 1: not meaningful to 7: very meaningful). Moreover, in order to guarantee variability in the answers, the original questionnaire was modified in the pilot version: 12 further items (FI) reporting errors (grammatical and/or semantic) were added to the items (OI) belonging to the original questionnaire. OI reported a mean score for each question >6 (almost the maximum); FI showed a mean score ≤1. These data confirmed that the content of the questionnaire was clear to the readers.

The population investigated was represented by eight-year-old children attending the third grade of primary school, selected through a cluster survey design, using the class as a sampling unit, a method also recommended by the WHO and widely used in international surveys [21,22]. First of all, the study was described to the enrolled children with regard to the objectives of the study and the instructions for survey completion. Additionally, parents were fully informed and provided their consent for study enrollment.

The questionnaire was composed of two macro-sections:

(a) Anthropometric form, in which a child’s weight and height were reported; measurement was performed at school by LHU healthcare professionals, previously calibrated. Weight was measured to the nearest 0.1 kg by an electronic balance. Children’s height was measured to the nearest 0.1 cm by a precision stadiometer;

(b) Children’s questionnaire. In the classroom, the children themselves completed the questionnaire, which was divided into three subsections asking:

*First section referring to the day of compilation:*

1. If they had breakfast;
2. If they ate at least one fruit or drank fruit juice for breakfast;
3. If they watched TV before going to school;
4. If they went to school on foot or by bicycle;
5. If they had a snack at school;
6. If they have eaten at least one fruit or fruit juice as a snack;
7. If they eat lunch in the school canteen.

*Second section referred to the previous afternoon:*

8. If they played video games, computers, tablets, or mobile phones;
9. If they watched TV programs;
10. If they played outdoors;
11. If they played sports.

*Third section referring to the previous evening:*

12. If they played video games, computers, tablets, or mobile phones after dinner;
13. If they watched TV after dinner;
14. If they brush their teeth after dinner.

Since the whole reference population included 3620 students, a sample of at least 348 individuals would have been required to investigate the selected variables, assuming a response proportion of 50%, a 95% confidence level, and a 5% margin of error.

The nutritional status in terms of obese/overweight and normal/underweight was compared between the two sex groups using the chi-squared test. A *p* ≤ 0.05 was considered statistically significant.

In order to evaluate the association between variables, a standard binary correlation matrix was used, showing correlation coefficients between variables (a score of 1 is the maximum association coefficient). A table was built in which each cell of the adopted matrix (crossing an abscissa and ordinate value) describes the level of correlation between the variables in abscissa and the variable in ordinate expressed as the Pearson’s correlation index, which is the ratio between the covariance and the standard deviations of each pair of variables.

### 2.2. Official Controls (OC) in Terms of Both Hygiene Conditions and Nutritional Guidelines

Parallel to the investigations directly conducted on the children, OC were carried out in school canteens. These OC were aimed at verifying both the hygienic–sanitary conditions and nutritional rules compliance. The controls were performed by a direct inspection of the canteens as well as by inspection of documentation completeness and maintenance [23,24].

The OC were performed by a team of official inspectors of the LHU: one medical doctor expert in human nutrition, one medical doctor expert in public health, one dietician, and one environmental health officer. The inspection team had to file a standardized report with one checklist, divided in two sections, provided by the Integrated Regional Control Plan to assess the elements of structural, procedural, and managerial compliance in the field of food safety in school catering (cooking centers, canteens with on-site preparation, and school refectories) [25]. The inspection was aimed at evaluating compliance with the requirements foreseen by law. In detail:

*Hygienic–sanitary section:*

1. Presence of accurate documentation held by the Food Business Operator (FBO) with respect to the activities carried out;
2. Presence of an adequate Hazard Analysis and Critical Control Points plan (HACCP);
3. Respect of status and hygienic conditions of systems, equipment, tools, premises, and structures;
4. Presence of raw materials, ingredients, and any other product intended for consumption;
5. Presence of semi-finished products, finished products and materials, and objects intended to come into contact with food;
6. Presence of disinfection procedures, ordinary and extraordinary cleaning, and maintenance;
7. Presence of production technological processes and transformation of food products;
8. Labeling, food products presentation, and preservation means, with particular attention to substances that cause food allergies and intolerances;
9. Previous non-compliance and corrective actions adopted;
10. Compliance with the regional guidelines for school and company catering.

As required by the guidelines for food safety OC, findings were recorded according to a four-point-scale that took into account full compliance (YES), partial compliance (yes), inadequacy/minor non-compliance (no) and major non-compliance (NO) [26].

*Nutritional section*:

1. Presence of tender specifications, canteen committee, and a plan for users who have allergies, intolerances, and/or adopt ethical/religious diets or diets adopted for non-health reasons;
2. Presence of a food safety training for kitchen and administration staff;
3. Presence of a nutritional table, menu validation, and correspondence between meals scheduled on the inspection day and the foods actually prepared; presence of allowed frozen or deep-frozen foods intended for preparations;
4. Presence of organic food and ingredients coming from a short supply chain;
5. Presence of IV or V range and/or canned foods;
6. Use of extra virgin olive oil and iodized salt;
7. Single- or multi-portion meal packaging;
8. Presence of a standardized plan for carrying out any food transport from external food establishments and transport time.

Regarding this section, the answers provided were dichotomous (yes/no).

### 2.3. Implementation of All the Prescribed Improvement Actions

Following the first cycle of OC and the actions and measures prescribed by the inspection team in case of non-compliance to official rules, follow-up OC were carried out to verify the implementation of corrective measures addressing the prescriptions given.

## 3. Results

### 3.1. Behaviors in Terms of Nutrition and Anthropometric Data

Overall, 26 classes from 22 different schools distributed across the provincial territory were enrolled. Of the 563 students attending the 26 classes enrolled, 501 completed the questionnaire (89%): 47.5% were male and 52.5% female, with an average age of 8.3 years (range 7–9 years). Weight status categories by sex are reported in Table 1.

Overall, 41.3% of children had a weight excess, which included both overweight and obesity. With regard to sex, males showed a lower percentage of overweight and normal weight but a higher percentage of obesity. Nevertheless, the statistical analysis did not show a significant association between sex and nutritional status.

**Figure 1:**
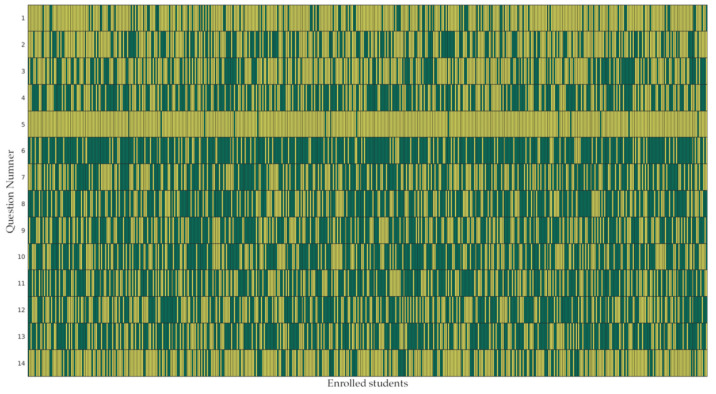
Answers distribution in the sample of students. The answer “NO” is represented in green, while the answer “YES” is represented in yellow.

In our sample, only 59.8% of children ate a qualitatively adequate breakfast that included at least one fruit or a fruit juice, and 10.8% did not have breakfast at all. Only 23.7% of the children consumed an adequate mid-morning snack, which included a fruit or fruit juice. Most children took an inadequate snack, and 2.8% did not consume one; 40.1% of the total children played outdoors the afternoon before the survey. Overall, 45% of children, on the morning of the survey, reported that they went to school on foot or by bicycle; conversely, 55% used a public or private means of transport. In our sample, 59.6% of children watched TV in the morning before going to school. Overall, 83.5% and 83.7% of children watched television or used video games/tablets/mobile phones the afternoon and the evening of the previous day, respectively. In addition, 71.5% of the children reported that they had brushed their teeth the evening before the survey. Answers to the fourteen questions are presented in Figure 1. Table 2 reports the results of the answers to the items of the questionnaire, stratified by nutritional status of enrolled children.

**Figure 2:**
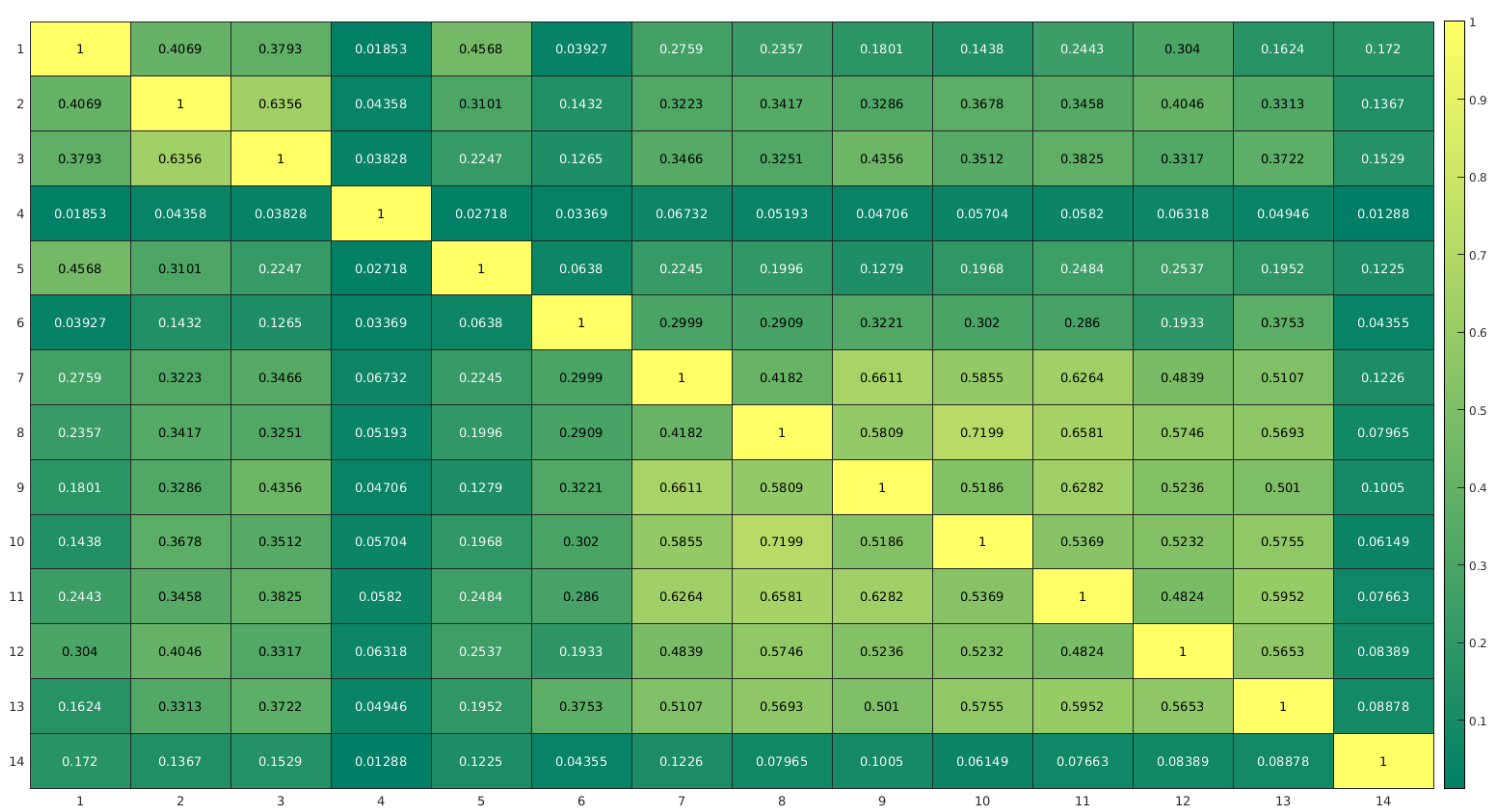
Correlation matrix showing the association between the answers to the questions numbered progressively in Table 2.

Figure 2 shows the correlation between the ratio of positive answers to the different questions in order to summarize data, and it gives a hint at possible clusters of co-occurring answers to groups of questions. Large values (close to 1) in this matrix indicate possible collinearity between the variables involved. Yellow cells show the maximum level of association between questions.

Our analysis suggests a mild correlation between the different children’s habits and lifestyles: the association between affirmative answers such as eating lunch at the school canteen correlates with the positive answers of playing sport, but it also correlates with playing video games, computers, tablets, or mobile phones and with watching TV programs. It also emerged that the morning breakfast with fruits or juices was done while watching TV. On the other hand, it is interesting to notice that “playing video games, computers, tablets, or mobile phones” were habits not associated with “going to school on foot or by bicycle”, analogously “play sport or play sport outdoor” was not associated with “going to school on foot or by bicycle”. It came to light that healthy family habits and proper lifestyles, according to our analytic model, may play an essential role in children’s health status.

**Figure 3:**
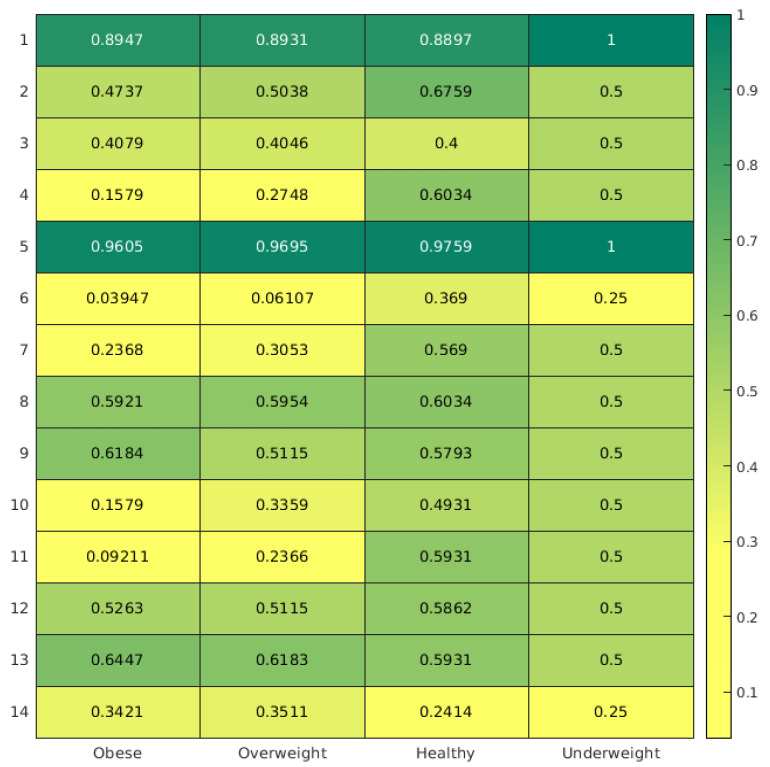
Correlation matrix showing the association between answers to the questions and nutritional status.

In Figure 3 is reported the correlation matrix between answers compatible with a healthier lifestyle and nutritional status. A yellowish color shows a possibly unhealthier lifestyle. As it is notable, the large part of the yellower cells is distributed among obese and overweight subclasses: in particular, unhealthy habits were registered with regard to question nos. 4, 6, 7, 10, 11, and 14 in the obese/overweight classes. On the contrary, the greener cells, indicating healthy habits, are more represented in the subclass of those with normal weight.

### 3.2. Official Controls (OC) in Terms of Both Hygiene Conditions and Nutritional Guidelines

Hygienic–sanitary and nutritional OC were carried out in the same 22 school complexes and 26 classes where students were enrolled for goal 1. Of the 22 schools, in 8 (36.4%) school canteens, meals were prepared on site, while 14 (63.6%) were managed by a specialized private company, and meals came from an external cooking center. A general compliance with the minimum requirements in terms of food safety was verified; however, some non-conformities were found in eight facilities. Some inadequacies were found only regarding the wear-out of some work-tops, the presence of mosquito nets that were not properly maintained, and the absence of lockers divided into dirty and clean compartments. Moreover, workspace areas were quite small with respect to the requirements (given for by the regional guidelines for school catering). In such cases, the Competent Authority issued mandatory provisions to the FBOs (Table 3—Hygienic–sanitary section).

A general compliance with the minimum requirements in terms of nutritional features was also confirmed, but in 27% of the enrolled facilities, an in-depth documentary investigation concerning the nutritional requirements of the menus proposed for special and non-special diets was needed. Furthermore, in 9% of cases, inconsistencies were found between the meals scheduled on the day of the OC and the foods actually prepared, due to the difficulty in finding on market and from retail sources the ingredients needed by the menu. The sample investigated also showed that 86% of FBOs have a specific certification of attendance to training courses on special diets, with particular reference to the methods of preparing and administering meals for people affected by celiac disease (Table 4—Nutritional section).

In total, for the hygienic–sanitary section, the number of prescriptions issued were as follows: documentary 1 (5%), structural 5 (23%), management 2 (9%), and organizational 3 (14%).

For the nutritional section, the number of actions that were taken to ensure full compliance with the sector regulations were, in particular: documentary 4 (27%), managerial 3 (14%), and organizational 2 (9%).

With regard to both nutritional and hygienic evaluations, no differences were reported between schools having internal or external services.

### 3.3. Implementation of All the Prescribed Improvement Actions

To allow the verification of compliance with the given prescriptions, eight follow-up OC were carried out during the four months following the first round of OC, aiming at verifying the resolution of 20 inadequacies and minor non-conformities previously detected in 8 of 22 schools enrolled. Despite some difficulties for FBOs related to finding the economic resources to be allocated to extraordinary maintenance interventions—which made it necessary to grant some time extensions to complete the execution of the works that involved structures or to encourage the purchase of materials—full compliance with the requirements and the checklists were concluded favorably during the scholastic year under analysis.

## 4. Discussion

The results of our study highlight a critical situation in our sample of eight-year-old students with regard to the percentages of overweight (26.1%) and obese (15.2%), which were greater than those detected at the national and regional level [3]. As a matter of fact, according to Italian surveillance, the national and regional levels of pathological obesity are 9.4% and 15.1%, respectively. The overweight percentages do not offer better data: 20.4% and 21.6% at national and regional level, respectively [3]. With regard to sex, although the differences among nutritional status are non-significant, in our study males showed a lower percentage of overweight vs females at the local level as well as at regional level, and males showed a higher percentage of obesity at the local level as well as at national level [3].

It has been confirmed that data concerning the population of children and adolescents with respect to obesity and overweight are alarming and represent one of the most serious public health problems of our time [27]. This situation is correlated with bad habits, such as the consumption of processed foods rich in simple sugars and fats, and with high calorie diets associated with a sedentary lifestyle and with the growth of mechanized transport, urbanization, and information technology [5,7,28]. With regard to incorrect habits, our sample, compared with national population, showed a high level of skipping breakfast (10.8% vs. 8.7%), low intake of fruits and vegetables (40.2% vs. 24.3%), and unhealthy snacking (76.3% vs. 55.2%) [3]. The latter habits have been associated not only with bad nutritional status but also with low cognitive performance [9]. With regard to physical activities, 55% of the sample went to school by walking or cycling (compared with 26.6% at the national level), and 40.1% in the afternoon watch TV or play videogames/tablet/cellphones (compared with 44.5% at the national level). Although the local situations seem better than those at national level, unhealthy habits were still frequent in our sample and need attention.

In our sample, healthy lifestyles and correct food habits were not always correlated, and also, while the consumption of fruit for breakfast was considerable, this habit was not related to playing sports or other activities outdoors. This is a very important aspect since a low level of physical activity in young students leads to a reduction in physical activity/sport practice experienced adults, highlighting the necessity of promoting sports in this school-age period of life [8]. Therefore, the implementation of targeted interventions of education and health promotion in primary schools can undoubtedly favor the spread of healthy habits, which represents, especially in children, a useful investment in the prevention of the development of NCDs.

With regard to the correlation matrix in Figure 3, it confirms that unhealthy habits are more common among students who are obese or overweight. However, it is particularly interesting to underline that eating at school was more common among students with normal weight and, on the contrary, that obese and overweight subclasses were associated with students who did not eat at school. Although this evidence needs to be studied in depth, it highlights the potential role that the school environments may play in health promotion to prevent nutritional disorders [9]. Consuming a nutritionally correct meal, one that is adequate to the needs of children and adolescents in the school context, may represent a qualitative and quantitative guarantee with respect to the energy needs of this target population: often the school canteen is the only time when the meal consumed meets the macro- and micronutrient needs of children. More studies are needed in order to further analyze and to set the canteen menus to the energy expenditure of children.

At the same time, in order to fulfill its health promotion task, the school canteen has to completely respect the official rules; therefore, OC during the management of food service at school are needed. An adequate and more effective OC planning could contribute to achieving better results in terms of the capabilities of the inspections performed and preventive interventions adopted, especially in school environments.

Previous data report that 58.9% of the schools have their own internal school canteen, and in 52% of cases, the menu drafting is carried out by LHU dieticians; in the remaining 48%, the menu is edited by external professionals [16]. In our experience, in only 36.4% of the cases are meals prepared within the school, and this aspect has pros and cons: the presence of an internal canteen favors meals that are produced on site and immediately served, which guarantees the organoleptic qualities, consistency, and minimal alteration of foods [15]. On the other hand, the external cooking centers, managed by large companies, guarantee standardized procedural aspects, but it is necessary to consider that the transport phase in food delivery bags has a few critical points (e.g., with respect to hot or cold chains) [15]. From our OC on food hygiene and nutritional safety, although there is substantial compliance with the regulatory requirements, some prescriptions aimed at conforming structural aspects were issued and, in some cases, it was necessary to investigate specific nutritional aspects at later stages. No difference was reported between schools having an internal or external food service. The constant and targeted control system for this type of activity is able to detect substantial and formal deficiencies and promote timely corrective actions, even potentially related to reducing the risks of foodborne diseases. Moreover, in our study, all the registered non-conformities were solved during the same scholastic year. This demonstrates that it is possible to obtain full compliance to the rules of law only by constant monitoring. The current local organization of OC can allow a single access made by a team of different healthcare professionals (medical doctor, dietician, environmental health officers, food technologist, etc.), each with a different training background, useful for creating favorable synergies with FBOs and for improving verification in the field, assessing both nutritional and hygienic–sanitary aspects jointly. To be completely efficient, these OC should also foresee laboratory test of environmental matrices and food, such as is done in other human environments [29].

To our knowledge, this is the first study reporting integrated data on children’s nutritional habits and OC in school canteens, which jointly investigated many aspects by different healthcare professional in a single inspection, an approach that is favored by regional reference laws, which are innovative in this regard [15,23].

The authors are aware of some limitations of the study. First, lifestyles were not investigated in depth, in order to avoid an excessive length of the questionnaire, the compilation of which could have favored rejection. This could have hidden important information, such as the children’s energy expenditure, as well as other sociodemographic variables that were not collected. Furthermore, this study was targeted to a sample of students attending schools that are not representative of the whole population of students in Italy. Therefore, our study can be considered as preliminary research. Due to the limitation in representativeness, further studies are needed to deepen the investigation in this subpopulation.

Furthermore, the study was conducted before the COVID-19 pandemic. Different studies have demonstrated that sedentary behaviors increased and that all physical activities decreased significantly during the lockdown [8]. Therefore, incorrect habits highlighted during the present study can be further worsened by the preventive measures adopted in response to the pandemic emergency. Since promoting physical activity during non-pandemic periods may also have positive effects in case of a lockdown [8], greater emphasis will have to be given to school interventions to promote healthy lifestyles, including those associated with OC conducted in school contexts. Finally, it should be noted that, in contrast with infectious diseases—where surveillance systems are already implemented for both health risk assessment and early detection also in critical situations [30,31]—continuous surveillance systems for risk factors of NCDs, such as overweight and physical inactivity, are very difficult to implement and maintain.

## 5. Conclusions

This study reports some critical issues regarding nutritional status and habits in eight-year-old students and some features of school canteen services. It is noteworthy to underline how eating at school was less frequent among obese and overweight students compared with those with normal weight. Although this evidence needs to be further confirmed, it highlights the contribution that the school canteens may provide for health promotion and prevention of nutritional disorders. On the other side, in order to fulfill its health promotion task, school canteens have to comply with official regulations and guidelines at every step of food chain; therefore, OC on school food management services are needed.

Data obtained from the present study may be useful in developing and implementing effective policies able to integrate nutrition education and OC for a healthier school environment.

## Figures and Tables

**Table 1:** Weight status categories by sex.

Range of BMI * Percentile	Total (*n*/%)	Female (*n*/%)	Male (*n*/%)
>95th percentile (obese)	76 (15.2%)	37 (14.1%)	39 (16.4%)
85th to 95th percentile (overweight)	131 (26.1%)	70 (26.6%)	61 (25.6%)
5th to 85th percentile (normal weight)	290 (57.9%)	153 (58.2%)	137 (57.6%)
<5th percentile (underweight)	4 (0.8%)	3 (1.1%)	1 (0.4%)

* BMI: body mass index.

**Table 2:** Answers provided by students, stratified by nutritional status.

Question		Total		Obese (BMI > 95th Percentile) (% of 501 People)		Overweight (BMI between 95th and 85th Percentile) (% of 501 People)		Normal Weight (BMI between 85th and 5th Percentile) (% of 501 People)		Underweight (BMI < 5th Percentile) (% of 501 People)	
	Nutritional Status/ Response to the Questions	Yes	No	Yes	No	Yes	No	Yes	No	Yes	No
*First section referring to the day of compilation*											
(1)	they had breakfast	447 (89.2%)	54 (10.8%)	68 (13.6%)	8 (1.6%)	117 (23.3%)	14 (2.8%)	258 (51.5%)	32 (6.4%)	4 (0.8%)	0 (0.0%)
(2)	they ate at least one fruit or juice for breakfast	300 (59.8%)	201 (40.2%)	36 (7.2%)	40 (8.0%)	66 (13.1%)	65 (13.0%)	196 (39.1%)	94 (18.8%)	2 (0.4%)	2 (0.4%)
(3)	before going to school they watched TV	299 (59.6%)	202 (40.4%)	45 (9.0%)	31 (6.2%)	78 (15.6%)	53 (10.6%)	174 (34.7%)	116 (23.1%)	2 (0.4%)	2 (0.4%)
(4)	they went to school on foot or by bicycle	225 (45%)	276 (55%)	12 (2.4%)	64 (12.8%)	36 (7.2%)	95 (19.0%)	175 (34.9%)	115 (22.9%)	2 (0.4%)	2 (0.4%)
(5)	they had a snack at school	487 (97.2%)	14 (2.8%)	73 (14.6%)	3 (0.6%)	127 (25.3%)	4 (0.8%)	283 (56.5%)	7 (1.4%)	4 (0.8%)	0 (0.0%)
(6)	they ate fruit or juice as a snack	119 (23.7%)	382 (76.3%)	3 (0.6%)	73 (14.6%)	8 (1.6%)	123 (24.5%)	107 (21.4%)	183 (36.5%)	1 (0.2%)	31, (0.6%)
(7)	they eat lunch in the school canteen	225 (45%)	276 (55%)	18 (3.6%)	58 (11.6%)	40 (8.0%)	91 (18.1%)	165 (32.9%)	125 (25.0%)	2 (0.4%)	2 (0.4%)
*Second section referring to the previous afternoon:*											
(8)	they played video games, computers, tablets, or mobile phones	201 (40.1%)	300 (59.9%)	31 (6.2%)	45 (9.0%)	53 (10.6%)	78 (15.6%)	115 (22.9%)	175 (34.9%)	2 (0.4%)	2 (0.4%)
(9)	they watched a program on TV	217 (43.4%)	284 (56.6%)	29 (5.8%)	47 (9.4%)	64 (12.8%)	67 (13.4%)	122 (24.3%)	168 (33.5%)	2 (0.4%)	2 (0.4%)
(10)	they played outdoors	201 (40.1%)	300 (59.9%)	12 (2.4%)	64 (12.8%)	44 (8.8%)	87 (17.4%)	143 (28.5%)	147 (29.3%)	2 (0.4%)	2 (0.4%)
(11)	they played sports	212 (42.3%)	289 (57.7%)	7 (1.4%)	69 (13.8%)	31 (6.2%)	100 (20.0%)	172 (34.3%)	118 (23.5%)	2 (0.4%)	2 (0.4%)
*Third section referring to the previous evening:*											
(12)	after dinner they played video games, computers, tablets, or mobile phones	222 (44.4%)	279 (55.6%)	36 (7.2%)	40 (8.0%)	64 (12.8%)	67 (13.4%)	120 (23.9%)	170 (33.9%)	2 (0.4%)	2 (0.4%)
(13)	after dinner they watched TV	197 (39.3%)	304 (60.7%)	27 (5.4%)	49 (9.8%)	50 (10.0%)	81 (16.2%)	118 (23.5%)	172 (34.3%)	2 (0.4%)	2 (0.4%)
(14)	after dinner they brushed their teeth	358 (71.5%)	143 (28.5%)	50 (10.0%)	26 (5.2%)	85 (16.9%)	46 (9.2%)	220 (43.9%)	70 (14.0%)	3 (0.6%)	1 (0.2%)

**Table 3:** OC—Hygienic–sanitary section (classified in 4 levels).

		YES	Yes	No	NO
(a)	congruity of documentation	22 (100%)	-	-	-
(b)	HACCP	21 (95%)	-	1 (5%)	-
(c)	hygienic conditions	20 (91%)	-	2 (9%)	-
(d)	raw materials and ingredients	22 (100%)	-	-	-
(e)	finished products and food contact materials	22 (100%)	-	-	-
(f)	disinfection, cleaning, and maintenance	17 (77%)	-	5 (23%)	-
(g)	production processes	22 (100%)	-	-	-
(h)	labeling	22 (100%)	-	-	-
(i)	resolution of previous non-conformities	22 (100%)	-	-	-
(j)	compliance regional guidelines for school catering	19 (86%)	-	3 (14%)	-

**Table 4:** OC—Nutritional section (classified in 2 levels).

		Compliant	Not Compliant
(a1)	tender specifications	22 (100%)	-
(a2)	canteen commission	22 (100%)	-
(a3)	plan for users with food allergies/intolerances/ethical–religious diets	22 (100%)	-
(b)	food safety training	19 (86%)	3 (14%)
(c1)	presence of nutritional table and menu validation	16 (73%)	4 (27%)
(c2)	correspondence of the meals scheduled and foods prepared	20 (91%)	2 (9%)
(c3)	presence of allowed frozen or deep-frozen ingredients	22 (100%)	-
(d)	presence of organic food/short supply chain	22 (100%)	-
(e)	presence of IV or V range and/or canned foods	22 (100%)	-
(f)	use of extra virgin olive oil and iodized salt	22 (100%)	-
(g)	single- or multi-portion packaging of the meal	22 (100%)	-
(h)	plan for external transport (if applicable)	14 (100%)	-

## Data Availability

The data presented in this study are available on request from the corresponding author. The data are not publicly available due to privacy and ethical reasons.
